# A cross-cultural analysis of spiritual transcendence and its impact on job satisfaction, job security, and life satisfaction in Bali and Türkiye: mediator effect of earthquake anxiety

**DOI:** 10.3389/fpsyg.2024.1402685

**Published:** 2024-07-31

**Authors:** Nyoman Sri Subawa, Elif Baykal, Ida Nyoman Basmantra, Caren Angellina Mimaki, Halil Yorulmaz

**Affiliations:** ^1^Faculty of Economics and Business, Universitas Pendidikan Nasional, Denpasar, Bali, Indonesia; ^2^School of Business and Management Sciences, İstanbul Medipol University, İstanbul, Türkiye; ^3^Research Center of Innovative Management, Azerbaijan State University of Economics (UNEC), Bakü, Azerbaijan; ^4^Vocational School, İstanbul Medipol University, İstanbul, Türkiye

**Keywords:** earthquake anxiety, job satisfaction, job security, life satisfaction, spiritual transcendence

## Abstract

Earthquakes are considered as a major factor causing PTSD, anxiety, and depression across various age groups. Increased anxiety stemming from earthquakes may prompt individuals to turn to spirituality as a coping mechanism, with spiritual transcendence believed to be an effective way to mitigate anxiety. In this study, Bali, which has a Far Eastern spiritual tradition, and Türkiye, the majority of whose population is Muslim, are discussed comparatively. In fact, the underlying reason for this choice is to examine whether there is a difference between Hindu belief, one of the Far Eastern religions as a spiritual tradition, and Islam, one of the monotheistic religions, regarding individuals’ ability to manage anxiety and some basic psychological reactions to the fear of earthquakes. An example of Bali’s Hindu tradition has been considered a representation of the Islamic tradition, one of the monotheistic religions in Türkiye. Given this phenomenon, the study took a quantitative approach, giving a novel conceptual framework for understanding the relationship between spiritual transcendence, seismic fear, job satisfaction, job security, life satisfaction, and the moderating influence of optimism. Empirical data were acquired via surveys issued via Google Form to a total of 913 workers in Bali and Türkiye. The research data were analyzed using SmartPLS software and a structural equation modeling technique. Findings indicate that earthquake anxiety and the impact of spiritual transcendence on satisfaction and job security are stronger in Bali than in Türkiye. Additionally, spirituality holds greater significance for the Balinese sample compared to the Turkish sample. The study clarifies the implications of its findings and provides guidance for future research endeavors.

## 1 Introduction

Kunii et al. (2022) report that anxiety levels are high in areas where natural disasters such as earthquakes occur, causing people to have problems in their daily lives and becoming increasingly anxious. Hence, it is popular among communities hit by natural disasters to study the connections between mental health issues, employment status, economic circumstances, and disasters (Katayanagi et al., 2020; Salawali et al., 2020; Khan et al., 2023). Actually, disasters like earthquakes make people feel scared, irritated, and anxious and make people worry about losing their lives, loved ones, and jobs (Bırni et al., 2024). An examination of statistical data indicates that Türkiye has experienced earthquakes roughly every five years, with a significant loss of life and property being caused by these natural disasters (AFAD, 2018) which make Türkiye a very vulnerable region for earthquakes.

On February 6, 2023, Türkiye was struck by two of the most devastating earthquakes in the last century (7.7 and 7.6 on the Richter scale). 41 thousand people have passed away and more than 115 thousand people had been injured (Sarı et al., 2023). Actually, in Türkiye these kinds of huge earthquakes occur constantly making people irritated and anxious. Similarly, Indonesia is among the nations with the most active seismic frequencies in the world due to its intricate geological layout. Because the Pacific Ring of Fire encircles Indonesia and stretches from Aceh, Sumatra, Java, Bali, Nusa Tenggara, and North Maluku to Papua, there have been numerous tectonic activities. For instance; on July 16, 2019, an earthquake with a magnitude of 6.2 occurred in southern Bali with deadly results affecting the local community. In fact, in both countries, earthquakes create a great deal of anxiety. Which made us perceive earthquake anxiety as a form of death anxiety. On the one hand, the two cultures compared in this study is chosen owing to the fact that they are considered as representatives of a culture with monotheistic religion (Islam) and Polytheistic religion (Hinduism). We wanted to see whether there is a profound difference regarding their effect on psychological reactions of individuals.

Within the topic of death anxiety, one of the most significant and prevalent theories is Terror Management Theory (TMT). According to Solomon et al. (2004), this theory assumes two barriers helping people tolerate death anxiety: self-esteem and a unique worldview that may include religious views. It makes sense to assume that both of these buffers are universal because they function similarly in every individual. TMT explains how religiosity could modulate death salience by acting as a defensive mechanism. The present study opted to apply the theoretical implications to earthquake anxiety in order to elucidate the impact of spiritual transcendence on life and satisfaction and job security, which we hypothesized to be less positively correlated with earthquake anxiety which is also some kind of anxiety related to death anxiety. Terror Management Theory recognizes worldview and self-esteem as the main obstacles to anxiety, but spiritual transcendence can also be employed as a third weapon in this context (Piotrowski et al., 2020).

Piedmont (1999) described spiritual transcendence as a person’s ability to see life from a broader, more objective perspective by stepping outside of their current sense of time and location and hence, in this study we assumed that this can affect the level of earthquake anxiety. In this study, we hypothesized that a person’s degree of life and job satisfaction may be adversely affected by the loss of resources and the anxiety that follow natural disasters. The individual’s level of spirituality plays a significant role in how they cope with life challenges. Those with a heightened spiritual perspective often reinterpret challenges as tests from a divine force, providing them with the strength to overcome adversities (Chakraborty and Sadachar, 2023). We base this on Hobfoll (1989) Conservation of Resources Stress Model. According to Hobfoll (1989) Conservation of Resources Model, people want to accumulate, hold onto, and safeguard their resources. When these resources are actually or supposedly lost, they cause stress.

However, the extent individuals experience spiritual transcendence can affect the way they perceive tragedies and adversities (Piotrowski et al., 2020). Natural disasters and other traumatic occurrences can frequently result in a large loss of resources, which has long-term detrimental effects on survivors’ physical and mental health (Zhang et al., 2022). Psychological stress can be caused by the threat of resource loss, loss of actual resources, or a lack of resource gain as a result of investing in one or more of the aforementioned types of resources (Hobfoll, 1989), leading to problems related to job and work satisfaction and can create a fear related to job security.

In Balinese context spiritual values are considered as one of the main pillars of social life (Rahmawati et al., 2019) whereas in Turkish context, at least in the last decades, spirituality has become a less significant phenomena in social life (Nişancı and Aysan, 2019). On the one hand, in general culturally Turkish society is known to be a very anxious society owing to economic problems and political turmoils making people psychologically exhausted and distracted (Karadağ and Sölpük, 2018) whereas, in Balinese case, we can witness a psychologically more resilient community (Djelantik et al., 2021). On the one hand, earthquake anxiety is widespread in both Türkiye and Bali (Tedjokusumo, 2023). Unfortunately, job security and job satisfaction remain low in Türkiye due to economic crises (Hidayat et al., 2023). Moreover, regarding life satisfaction, we come across descending life satisfaction levels in Türkiye (Güler et al., 2024) and rather peaceful atmosphere leading to life satisfaction in Balinese context (Muliawati and Puspawati, 2023). Taking into consideration the possible effects of spiritual transcendence on earthquake anxiety, we attempted to test whether earthquake anxiety is effective as a mediator in the relationship between spiritual transcendence on job and life satisfaction levels of employees and effect their sense of job security in two different cultures, Turkish and Balinese culture. As an additional objective, we aimed to explore the possible moderator effect of optimism in the relationships between earthquake anxiety and satisfaction levels and sense of security.

## 2 Literature review

### 2.1 Spiritual transcendence

The concept of spirituality closely related to the concept of religion, yet there are a clear difference between the two terms (Hirono and Blake, 2017; Bauer and Johnson, 2019; Lace et al., 2020). Spirituality can be described as a psychological process experienced by individuals that involves self-transcendence, increased awareness, and the creation of a relationship with a sense of divinity or sublimity which then becomes the underlying aspect of the individual’s beliefs, identity and actions (King et al., 2020). Furthermore, Shabani et al. (2023) argue that spirituality includes several components, namely an individual’s understanding of the purpose of life, awareness of life values, transcendence capacity, as well as the individual’s ability to connect with oneself, other people, nature, as well as higher power.

Ekşi et al. (2020) also believed that spirituality can be reflected through the process of searching for the meaning of life, transcendence, modesty, achieving high values, as well as the process of discovering the secrets of life. Spirituality can also be defined as values, attitudes, or hopes that connect a person to well-being, so that it is considered very essential in creating happiness, increasing understanding of the meaning and purpose of life, and can lead to achieving life satisfaction (Jadidi et al., 2022; Kasapoğlu, 2022). As to Piotrowski et al. (2020), spirituality also covers some kind of transcendence that can be described as an individual’s self-awareness and connection to an ultimate source outside the self, especially God, which gives meaning to their life.

Furthermore, Piedmont (1999) defined spiritual transcendence as the ability that individuals have to view and understand life from a broader or higher perspective and more objectively, especially related to the individual’s understanding of the synchronicity of life, awareness of everything interconnected, as well as awareness to develop a sense of commitment toward other individuals. Spiritual transcendence scale consists of three main components: connectedness, which emphasizes a sense of connection and awareness of the importance of creating long-term harmony in life, universality, which emphasizes a strong belief in the unitary nature of life, and prayer fulfilment, which refers to a sense of joy and satisfaction that results from a personal encounter with transcendent reality.

In the process of self-transcendence, individuals tend to carry out various activities whose main aim is to help other people in need, as well as share wisdom with other individuals. Soriano and Calong (2021) argue that self-transcendence leads to the practice of increasing spiritual well-being in the midst of difficult conditions, especially those related to suffering, where individuals who experience these difficulties tend to share wisdom, accept death as a process of life, are interested in helping others, let go of loss, and find spiritual meaning in life. This can also be strengthened by studies which reveal that individuals with a high level of spirituality will be more patient in facing life’s challenges, especially when they lose someone (Nejati-Zarnaqi et al., 2022).

In addition, individuals will feel a strong awareness regarding God’s great spiritual plan which will be able to bring them to growth so they will tend to accept all events that occur as a process of growth (Khursheed and Shahnawaz, 2020). In this case, individuals with a high level of self-transcendence will better understand and accept difficult situations that are beyond their control, especially those related to severe traumas or natural disasters. Most people often use the spirituality principles in dealing with the various life challenges they experience, where a high level of spirituality tends to encourage individuals to re-evaluate the challenges they experience as a test given by God, which then actually gives them the strength to overcome these challenges (Chakraborty and Sadachar, 2023).

Spirituality is believed to be the main contributor that can influence an individual’s level of satisfaction with their life as a whole, especially in dealing with and managing stress, anxiety and depression so that this can lead to increased productivity (Shabani et al., 2023). In this case, spirituality can be said to be a crucial element for human life. Margetić et al. (2022) in their study revealed that solving problems through spiritual principles can be a significant foundation for negative emotions that arise from the surrounding environment. Literature reveals that aspects of spirituality, especially transcendence and humanity, provide a strong correlation to post-traumatic growth during the COVID-19 pandemic (Casali et al., 2022). The successful impact of the pandemic has triggered high levels of stress among individuals, where they will choose to draw closer to God, utilize spiritual ties, and ask for Divine guidance when facing vulnerabilities in life (Chakraborty and Sadachar, 2023).

Related to this, prayer is a strong spiritual source and can be used as a means of expressing individual psychological dynamics, as a spiritual tool for dealing with suffering, access to holiness, and a means of self-transcendence (Esperandio and Ladd, 2015). Research conducted by King et al. (2020) also demonstrated that high levels of religiosity reflect high levels of personal spirituality, which then leads to higher levels of hope. In this case, individuals tend to show a high sense of belonging, a perception that God is the most important, and a high commitment to their beliefs. Furthermore, the findings in the study conducted by Ekşi et al. (2020) demonstrated that spiritual transcendence can lead individuals to happiness, especially by asking a help from God.

### 2.2 Spiritual transcendence in Balinese culture

Bali, is one of the regions in Indonesia, which is known to have a high level of spirituality or can be said to be the center of world spiritual energy. One of the philosophies that is strongly held by the people of Bali is Tri Hita Karana, namely the responsibility to maintain a balanced relationship between environmental, social and spiritual aspects which is also a distinctive characteristic of Balinese culture (Warren, 2012). Through this philosophy, the Balinese people are committed to maintaining spirituality by always serving God, protecting nature, while maintaining harmony with fellow humans (Rosalina et al., 2023). Moreover, the Balinese people also have strong local wisdom, especially in dealing with natural disasters. Balinese people believe that the occurrence of natural disasters is a sign of disharmony in human life and the occurrence of natural disasters is a form of God’s anger due to human actions that do not protect the world properly (Paramita, 2018).

Thus, the spiritual activities carried out by people in Bali are aimed at regenerating and maintaining balance in the universe. Nejati-Zarnaqi et al. (2022) in their research succeeded in proving that individuals perceive disasters as an inseparable part of this world, where natural disasters occur as a form of test given by God so that humans can grow into adults, perfect, trained, strong, able to expand their potential, and able to cultivate a deep relationship with God. In this context, natural disasters and other forms of suffering such as disease and destruction are one of the paths given by God to the world, especially in spiritual growth. Thus, it can be confirmed that spirituality is essential element of healing and growth in times of trauma and post-disasters (Dueck and Byron, 2011; Van Cappellen et al., 2021).

### 2.3 Spiritual transcendence in Turkish culture

In the historical process, the Muslim people living in the Ottoman society, which formed the foundations of the Turkish society settled in Türkiye, adopted the values of a cultural atmosphere in which belief in the afterlife predominated, like many societies based on agricultural production and living a monotheistic religious life (Erkol, 2015). Until the 19th century, there was a society in which differences lived together as a result of geographical conditions within the Ottoman society (Türköne, 2003). The most important element that protects this structure was religion. In modern Türkiye, spirituality is equated with conservatism (Baykal, 2021).

The emergence and development of conservatism in Türkiye is closely related to the experience of modernization. The 70s appear to be a turning point regarding the increase in the visibility of religion in Turkish society (Asanatuci, 2023). Despite the experience of modernization in Türkiye, religion found a place for itself in the general social structure with new forms and managed to continue its existence at the social and individual level, and appropriated many elements of modernity by melting them in the crucible of tradition. In other words, the plurality and multidimensionality of Islamic identity claims do not form an anti-modern discourse about religious self, but rather a politics of identity acting within modernity and seeking respect (Keyman, 2007).

According to Islamic belief accepted by Turks, the tendency to take refuge in Allah and to show patience in the face of difficulties by getting support from Him can contribute to the individual feeling strong and coping with difficulties, and maturing by facing the fact that there is pain in life (Gümrükçüoğlu, 2023). In the divine book, the Qur’an, patience means moving forward with firm and determined steps, without panic or haste, in case of encountering troubles and difficulties, and waiting for God’s will and judgment. In this sense, prophet Muhammad advised the believer to stand firm in the face of calamities and disasters and to survive without collapsing.

### 2.4 Earthquake anxiety

Natural catastrophes such as floods, hurricanes, typhoons, and tsunamis, which have resulted in numerous casualties and infrastructure damage, are among the most feared by everyone on the planet. Earthquakes are among the most destructive natural disasters, and their occurrence is impossible to forecast. The earthquake tragedy is believed to be able to cause posttraumatic stress disorder (PTSD), anxiety, and even depression in all age groups (Mesidor and Sly, 2019). The literature reveals that anxiety is one of the components that usually appears after a natural disaster, especially an earthquake (Cénat et al., 2020; Gerstner et al., 2021). The results of research conducted by Fan et al. (2011) in China proved occurrence of anxiety, depression or PTSD even a six months after the earthquake incident.

Similarly, Xi et al. (2020) identified that people living in disaster-prone areas such as the Sichuan region were proven to have very high levels of PTSD, which also contributed to the high levels of depression and anxiety symptoms experienced. The same relationship was also found by the results of Pistoia et al. (2018) study which revealed that individuals who were in areas that were more frequently affected by natural disasters, especially earthquakes, tended to have high levels of anxiety and were more alert to threats. Hogg et al. (2016) argued that moving to another location that is safer and less frequently affected by disasters such as earthquakes may act as a post-earthquake protective effect that can offer better living conditions by reducing stress levels, trauma, depression or anxiety.

Goenjian et al. (2000) identified that the earthquake phenomenon had succeeded in triggering the creation of traumatic experiences in individuals, especially adults, where this severe trauma was caused by strong memories of conditions after the earthquake such as seeing many destroyed buildings, resulting in a reduction in community members either due to death or moving to other areas. Supporting this, Mutianingsih and Panjaitan (2020) stated that elderly individuals are more likely to suffer from post-disaster psychological problems such as anxiety, depression and PTSD which are believed to have a negative influence on the well-being and health of elderly victims. In this case, the increasingly severe level of depression was caused by the many pressures and difficulties experienced by adults after the earthquake, such as poverty, lack of food supply, and difficulty in finding work, so this had an impact on challenges in overcoming the trauma experienced. Furthermore, the increasingly severe level of anxiety is also driven by a number of factors, one of which is the great fear of a recurrence of the phenomena experienced during and after the earthquake (Mutianingsih and Panjaitan, 2020). Similarly, Zhang et al. (2012) in their research revealed that elderly individuals who had experienced a devastating earthquake were very likely to suffer from psychological problems such as a tendency to suffer from PTSD, depression and high levels of anxiety even more than 1 year after the natural disaster. On the one hand, the study conducted by Tang et al. (2020) stated that teenagers continue to face quite high anxiety problems even though an earthquake occurred three years ago, where the research results reveal that the serious challenges that must be faced after the earthquake are a critical factor in teenage anxiety. Türkiye is an earthquake zone where important fault lines pass (Aral and Tunç, 2021). In Türkiye, earthquake reality is a phenomenon making people very uneasy. Many people experience psychological problems due to earthquake anxiety or lose their jobs, physical fitness or mental health because they have actually experienced the earthquake disaster (Aydınbaş, 2023). Similarly, volcanic earthquakes are quite common in Bali and make the local people uneasy (Ardianto et al., 2021). However different from Turkish case, in Bali, earthquake anxiety is coming after anxiety stemming from floods caused by heavy rain (Subadra, 2020; Sutarja et al., 2022).

In this study, we assumed that earthquake anxiety can be seen between earthquake anxiety and some possible psychological reactions like sense of job security, job satisfaction and life satisfaction. In previous studies it was revealed that in both Balinese context and Turkish context earthquake anxiety negatively effects life satisfaction (Djelantik et al., 2021; Güler et al., 2024). Similarly, job security and satisfaction seems to get lower in times of disasters in both cultures (Basmantra et al., 2023; Sert et al., 2023; Güler et al., 2024). Moreover, as Ardhana (2020) suggest in terms of optimism in community Balinese culture is highly positive while as Öcal et al. (2020) suggest Turkish culture is rather pessimistic.

### 2.5 Hypotheses

#### 2.5.1 Spiritual transcendence on earthquake anxiety

According to Walsh (2020) research, disasters cause considerable losses and numerous fatalities. As mentioned before, the presence of a disaster that occurs is also believed to be able to give rise to mental problems such as trauma, stress, depression, and excessive anxiety or fear of a repeat of the disaster experienced. As to the extant literature, there are various types of strategies that can be used by individuals when experiencing suffering, trauma, depression, anxiety or stress caused by disasters (Mesidor and Sly, 2019). For instance, Walsh (2020) in his study, argued that spiritual values and transcendent relationships are one of the most effective strategies in supporting healing of suffering experienced after a disaster, whether short-term or long-term at the same time.

Furthermore, he also explained that transcendent values and practices help individuals to survive and recover from the suffering they experience, especially when the individual cultivates meaning, connection, harmony and aligning goals. Jibeen et al. (2018) explored the relationship between spiritual transcendence and psychology in the psychology of natural disaster victims. They discovered that a high level of spiritual transcendence shields people from the negative impacts of natural disasters such as trauma, pressure, stress, depression, or anxiety, meaning that the presence of transcendence spirituality contributes to good changes in individual psychology. According to Pargament and Saunders (2007), those who pursue high levels of spirituality have a better quality of life because they are less likely to suffer from depression, anxiety, stress, or other psychological issues.

Aten et al. (2014) also discovered that religion and spirituality play a significant part in the psychological recovery process for the vast majority of disaster survivors. This person’s spiritual belief will aid them in recovering from traumatic occurrences and can be used to avoid negative consequences, particularly to their health. This is also supported by research results Fahm (2019) which demonstrated that one of the main factors that drives the emergence of fear and anxiety in individuals, especially when natural disasters occur, is a lack of psycho-socio-spiritual preparation. According to Fahm this state of spirituality can be built by boosting awareness and trust in the presence of God who always provides protection for individuals so that ultimately reduces fear and anxiety.

*H1*: Spiritual transcendence has a negative relationship toward earthquake anxiety.

#### 2.5.2 Spiritual transcendence on job security

Job security ought to be understood as an individual’s perceptions of the immediate work environment (Hur, 2022). Job security benefits employees because it promotes economic stability, allows people to plan their own futures, boosts self-esteem, and establishes order Interestingly, it improves employees’ mental health and sends positive signals to the brain, reducing mental stress and promoting work-life balance (Baykal et al., 2023).

On the one hand, when people experience spiritual transcendence, they regard the world as more important and worthwhile to experience. This also applies to their work life (Probst and Strand, 2010). In their empirical study Hamidipour and Rajabi (2018) found a meaningful relationship between the social security feeling and spiritual level, spiritual attitude and spiritual ability. As to Hamidipour and Rajabi (2018), it is possible to enhance sense of security by promoting spirituality and cultural intelligence. When employees feel spiritually more powerful they tend to become more inclined to perceive life positive (Hanin Hamjah et al., 2020). This positive mindset has the potential to give greater sense of job security (Pipera and Evangelia, 2021). Hence, being inspired from these studies, the second hypothesis has been created:

*H2*: Spiritual transcendence has a negative relationship toward Job Security.

#### 2.5.3 Earthquake anxiety as mediator between spiritual transcendence and job security

According to Hobfoll’s Conservation of Resources theory (1989), earnings and employment security are both significant resources in life, and their loss can generate (extra) stress and have a detrimental impact on well-being. Unemployment is defined as the loss of resources from all of Hobfoll’s categories, namely object resources (such as a car and a house), condition resources (such as employment and marriage), personal resources (such as skills and self-esteem), and energy resources (such as money and knowledge). In broad terms, substantial natural disasters such as the Tangshan earthquake (1976), the Katrina disaster (2005), and the Haiti earthquake (2010) had far-reaching consequences, including the (partial) destruction of local factories and other commercial and non-commercial businesses (van der Velden et al., 2023). Similarly, after earthquakes occurred in El Salvador in 2001, 32,540 people lost their jobs.

According to the extant literature, some adverse life events may result in lower levels of job security. Research conducted by Nandi et al. (2004) revealed that the experience of traumatic events such as natural disasters or acts of terror has given some individuals a stress response, for example the September 11 attacks in New York have made almost a third of people experience high levels of trauma and fear of negative impacts on their work, such as losing work time, experiencing layoffs, and others. Similarly, Groen et al. (2020) discovered that Katrina had a short-run negative impact on average earnings, driven by increasing unemployment, while the (seven-year) long-run effect was to improve earnings, reflecting increased labor demand.

For instance; Lin et al. (2021) discovered that during Covid-19, many persons had high levels of job insecurity due to pandemic-related worries. Pandemics can be considered among severe adversities that can give rise to anxieties about job security. However, disasters are at the top of the list that create irritation for employees and make them feel uncomfortable with their job security. In the extant literature there are myriad proofs for the negative effect of disasters on job insecurity (Kirchberger, 2017; Ardebili and Banisi, 2020). People even think about leaving their jobs and homes when they feel the risk of earthquakes (Jansen et al., 2017). Hence, in this study we wanted to test whether this powerful actor, earthquake anxiety, has a mediator effect in the relationship between spiritual transcendence and job security:

*H3*: Earthquake anxiety acts as a mediator in the relationship between Spiritual Transcendence and Job Security.

#### 2.5.4 Spiritual transcendence on life satisfaction

In Piedmont (1999) definition of spiritual transcendence, spiritual transcendence is “The ability of individuals to stand outside of their immediate sense of time and place to view life from a larger, more objective perspective.” It is a universal personality feature shared by individuals worldwide (Piotrowski et al., 2019). Spiritual transcendence is a multifaceted personality trait that includes prayer fulfilment, the ability to create a personal space in which one can feel a positive connection to some larger reality; universality, the belief in a larger meaning and purpose to life; and connectedness, feelings of belonging and responsibility to a larger human reality that transcends future generations and audiences (Piotrowski et al., 2020) that make individuals understand and enjoy the essence of life better.

Similarly, according to Rowatt et al. (2006), spiritual transcendence is positively correlated with self-reported humility, which can be viewed as the essence of an individual’s satisfaction with their own lives. Spiritual transcendence represents the ability “to rise above suffering and hardship through larger values, spiritual beliefs and practices, and experiencing transformations in new priorities, a sense of purpose, and deeper bonds” (Walsh, 2020). It is a powerful coping strategy in the face of adversity (Dorais and Gutierrez, 2021) making it easier to tolerate adversities of life and enjoy it. Being inspired from the extant literature, we hypothesized that:

*H4*: Spiritual transcendence has a positive relationship toward life satisfaction.

#### 2.5.5 Earthquake anxiety as a mediator between spiritual transcendence and life satisfaction

Earthquakes also have significant effects on mental states of individuals. Actually, psychological reactions to earthquakes occur in three stages. During the first stage, the Heroic phase, as adrenaline levels rise, people become more capable of coordinating help from strangers, assisting others, and saving themselves and their families. The honeymoon phase is defined by optimism and the assumption that everything would return to normal, if not better, after the calamity. It usually lasts two to four weeks. The third phase, which often begins in the second or third month after the disaster and lasts up to 36 months and includes dread of recurrence of the earthquake, is when the largest risk of psychological diseases emerging (Gerstner et al., 2021).

Actually, in this phase individuals are more prone to anxiety and their life satisfaction is quite low. Previous research showed the possible effects of disasters on mental states of individuals (Morganstein and Ursano, 2020; Mao and Agyapong, 2021). More specifically, earthquakes also have detrimental effects on psychological health of individuals (Cénat et al., 2020; Valladares-Garrido et al., 2022). Hence, we assumed that earthquake anxiety may shadow the positive effect of spiritual transcendence on life satisfaction. Hence, in this study we assumed that earthquake anxiety can act as a mediator in the relationship between spiritual transcendence and life satisfaction.

*H5*: Earthquake anxiety can act as a mediator in the relationship between spiritual transcendence and life satisfaction.

#### 2.5.6 Spiritual transcendence on job satisfaction

The congruence of corporate and employee objectives and values is a crucial facet of spirituality in the workplace (Baykal, 2024). According to spirituality researchers, spiritual transcendence can lead to a strong sense of alignment of their purposes with organizational goals contributing to meaning at work (Milliman et al., 2003). Actually, spirituality at work can result in higher integration, serving as a means to make individuals engage in their jobs with both their souls and minds leading to job satisfaction (Narcikara, 2017). As a matter of fact, Individual level spirituality contributes to perception of greater job satisfaction (Rashidin et al., 2020; Prihandono and Wijayanto, 2021).

Spiritual transcendence explains the human capacity to shift their awareness into a larger paradigm making things more understandable (Dorais and Gutierrez, 2021). It creates clarity of vision and perception making one’s job considered by the individual as more meaningful (Narcikara, 2017). Similarly, Widodo and Suryosukmono (2021) suggest spiritual transcendence creates a positive perception regarding meaningful work that has the potential to give birth to greater levels of job satisfaction. In previous studies, we can see empirical examples of positive effects of spiritual transcendence on job satisfaction (Chawla and Guda, 2010; Prihandono and Wijayanto, 2021). Being inspired from the extant literature, we hypothesized that:

*H6*: Spiritual transcendence has a positive relationship toward job satisfaction.

#### 2.5.7 Earthquake anxiety as a mediator in the relationship between spiritual transcendence on job satisfaction

As to Nagata, Tateishi and Mori, Professionals who have to continue working actively after severe natural disasters may experience post-traumatic stress disorder and resulting unhappiness, restlessness and poor performance in the work environment. Xi et al. (2020); Xu and Wu (2014) noted in their study that the anxiety caused by natural disasters, one of which is earthquakes, can create extra stress to individuals, which has been shown to influence their job satisfaction. The research results demonstrate that stress due to the fear of an increasingly severe earthquake is proven to be able to influence an individual’s mood at work, where individuals tend to feel pressure due to an earthquake which has damaged the previously established work balance and can ultimately have an impact on worsening the work environment. Being inspired from the extant literature, we hypothesized that earthquake anxiety can act as a mediator in the relationship between spiritual transcendence and job satisfaction in a negative direction shadowing the possible positive affect of spiritual transcendence on job satisfaction.

*H7*: Earthquake anxiety acts as a mediator in the relationship between spiritual transcendence and job satisfaction.

#### 2.5.8 Optimism as moderator

According to positive psychology literature, when confronted with difficult situations, optimistic people tend to generate more positive feelings, resulting in a greater sense of well-being, whereas pessimistic people produce more negative feelings (Carver and Vargas, 2011) and more positive reactions. Supporting this view, Supervía et al. (2020) revealed important statistical relationships between optimism-related variables, goal orientation and life satisfaction. In the extant literature, various empirical studies revealed that optimism is negatively related to psychological symptoms such as depression (Hirsch et al., 2007), stress (Flora and Lissy, 2021) and negative religious coping (Warren et al., 2015), and is positively related to life satisfaction (Warren et al., 2015), job satisfaction (Zhang et al., 2020) and positive religious coping (Warren et al., 2015).

Actually, optimism has acted as a moderator in many previous studies testing effects of various variables on life satisfaction such as Chen et al. (2016) and Gungor et al. (2021), job satisfaction but there is no study related to job security. Actually, optimisms make people expect good things to happen when faced with difficulty (Carver and Vargas, 2011) and buffer the impacts of negative life events. Hence, in this study, we chose to consider whether or not optimism, besides increasing these positive variables, would act as a moderator between earthquake anxiety and outcomes like life satisfaction, job satisfaction and perception of job security. Hence, we created the following hypotheses:

*H8*: Optimism act as a moderator between earthquake anxiety and perceived job security.

*H9*: Optimism act as a moderator between earthquake anxiety and life satisfaction.

*H10*: Optimism act as a moderator between earthquake anxiety and job satisfaction.

## 3 Method

### 3.1 Research design

This research employed a quantitative approach to explore deeply into the relationships among spiritual transcendence, earthquake anxiety, job satisfaction, job security, life satisfaction, and the moderating role of optimism. Conducted in two distinct locations, namely Bali and Türkiye, the study was motivated by the frequent occurrence of natural earthquakes in the former and the robust spiritual foundation shared by both regions. The data collection process was carried our between October 2023 and January 2024. In this study, in terms of cultural difference we assumed a possible difference between the religious perspectives of these two cultures and in relation to that we assumed it will create some differences between psychological reactions regarding earthquake anxiety. We did not have and did not mention a broad cultural analysis comparing the two cultures in all domains of life. The primary objective of this study was to contribute to the existing literature by offering a comparative analysis of the levels of spiritualism and its correlation with earthquake anxiety, job satisfaction, job security, life satisfaction, and optimism across these two diverse locations.

### 3.2 Participants

The target population included all workers residing in Bali and Türkiye. The determination of the sample in this study was carried out through purposive sampling approach, by setting specific criteria: female and male workers residing in the regions of Bali and Türkiye, aged at least 18 years old, and employed in either the private or public sectors. The utilization of purposive sampling allowed the researchers to target individuals who met the defined criteria, ensuring that the sample represented workers who had direct experience with earthquake events while at work. The rationale for selecting workers as research respondents was to understand how earthquake-related anxiety can influence job security and satisfaction. Data collection was conducted through the distribution of 10-point Likert scale questionnaires using the Google Forms platform for its practical and efficient method to gather empirical data. The final sample size of 913 respondents, evenly distributed between Bali (*N* = 457) and Türkiye (*N* = 456). According to gender, the study found that female workers dominated the participation, totaling 501 respondents, while male workers comprised 412 participants. Regarding the sector of employment, 594 respondents worked in the private sector, whereas 319 were employed in the public sector. Respondents’ ages were categorized into five groups: 18–25 years (*N* = 255), 26–35 years (*N* = 253), 36–45 years (*N* = 228), 46–55 years (*N* = 112), and > 56 years (*N* = 65). In terms of educational level, respondents were classified into four categories: high school graduates (*N* = 134), Bachelor’s degree holders (*N* = 320), Master’s degree holders (*N* = 133), and doctoral degree holders (*N* = 326).

### 3.3 Measures

In this study in order to measure spiritual transcendence, Turkish adaptation of Piedmont (1999) Spiritual Transcendence Scale, created by İme et al. (2019), was employed. In its original form, this adaption consists of 5 dimensions: spiritual transcendence, spiritual orientation, worship satisfaction, internationality, and connectivity. For this study, we focused on the first dimension, spiritual transcendence, with 9 items. For earthquake anxiety, we used the one dimension-seven item adaptation of Fear of Earthquake Scale (FES) developed by Prizmić-Larsen et al. (2023) to Turkish by Usta et al. (2024). Moreover, for optimism we used The Turkish adaptation of Luthans et al. (2007). Psychological capital scale, conducted by Akçay (2014). The optimism dimension was composed of 6 items. Furthermore, to measure the sense of job security we used one dimension-6 item job security scale developed by Yilmaz and Aydin (2023). For measuring life satisfaction, Turkish adaptation of Diener et al. (1985) life satisfaction scale conducted by Dağlı and Baysal (2017) has been used. This scale was composed of one item and five questions. In this study, in order to measure job satisfaction, the short form of the Job Satisfaction Scale, which was developed by Brayfield and Rothe (1951) and whose 5-item short form was created by Judge et al. (1998), was adapted into Turkish by Keser and Bilir (2019).

### 3.4 Data analysis procedures

The gathered data was subsequently analyzed using Structural Equation Modeling (SEM) with SmartPLS software. The data analysis involved two testing procedures: PLS Algorithm and PLS Bootstrapping. In the initial stage, the data was analyzed to ensure validity and reliability through outer model measurement. Convergent validity was assessed by examining outer loadings and Average Variance Extracted (AVE) value, where should ideally surpass a threshold of 0.50 to establish robust measurement (Hair et al., 2021). To ascertain discriminant validity, current study utilized the Fornell-Larcker Criterion and also employ the Heterotrait-Monotrait (HTMT) Ratio which should not exceed 0.90 to establish distinctiveness between constructs (Ringle et al., 2023). The use of Fornell-Larcker Criterion serves to investigate inter-construct correlations, primarily through the assessment of root squares of AVE. Subsequently, findings are cross-validated using HTMT Ratios which offer higher sensitivity in identifying correlations between constructs, thus enhancing the accuracy and validity of research data (Henseler et al., 2015; Henseler, 2017). Reliability of the data was evaluated using Cronbach’s Alpha and Composite Reliability, with scores exceeding 0.70 indicating reliability. The second stage involved employing PLS Bootstrapping procedures, which included Goodness of Fit analysis (R^2^) and hypothesis testing. This phase aimed to understand how spiritual transcendence in both regions could influence earthquake impacts and its relationships with constructs such as job security, job satisfaction, and life satisfaction. Additionally, it was crucial to investigate how levels of individual optimism, influenced by diverse cultural backgrounds, could impact earthquake-related anxieties.

## 4 Results

### 4.1 Validity assessment

The study gathered data through online questionnaires distributed to participants residing in Bali and Türkiye. The collected data was subsequently analyzed separately using Structural Equation Modeling (SEM) to derive results enabling thorough comparisons. The analysis comprised two stages: First, Outer model testing, which involved assessing convergent validity using outer loading parameters and *Average Variance Extracted (AVE)* values, discriminant validity using *Fornell-Larcker Criterion* and *Heterotrait-Monotrait (HTMT) ratio*, and reliability measures including *Cronbach’s Alpha* and *Composite Reliability*. Second, Inner model evaluation, which included testing the goodness-of-fit (*R^2^*) and hypothesis testing (Hair et al., 2021).

Tables 1, 2 presents the outcomes of the validity tests for the datasets from Bali and Türkiye, evaluated through outer loading and *Average Variance Extracted (AVE)*. The validity assessment revealed that not all indicators can be considered valid. According to Bali dataset (Table 1), four indicators (JSAT5, JSEC3, OPT4, and SPT9) were deleted and in the Türkiye dataset (Table 2), five indicators (JSAT3, JSAT5, OPT4, SPT4, and SPT9) were excluded due to their outer loading values below 0.50. Additionally, the *AVE* values before the deletion of these indicators were below 0.50: specifically, the spiritual transcendence variable scored 0.472 in the Bali dataset and 0.442 in the Türkiye dataset. Moreover, the optimism variable also scored below 0.50 which is 0.474 in the Türkiye dataset. Elimination of indicators in particular construct was carried out considering that the outer loading value obtained was relatively weak, namely below a score of 0.40 (Hair et al., 2021). After eliminating the invalid indicators, it confirms that both datasets were confirmed to meet the validity requirements: all outer loading values were above 0.50, and all *AVE* values for each variable were above 0.5. Specifically, the *AVE* values in the Bali dataset ranged from 0.524 to 0.741, and in the Türkiye dataset, they ranged from 0.562 to 0.844.

**Table 1:** Validity test results for Bali dataset (*N* = 457).

Variables	Indicators	Before indicators deletion		After indicators deletion	
		Outer loading (≥ 0.50 Valid)	AVE (≥ 0.50 valid)	Outer loading (≥ 0.50 valid)	AVE (≥ 0.50 valid)
Earthquake anxiety (EA)	EA1	0.750	0.689	0.752	0.689
	EA2	0.848		0.849	
	EA3	0.857		0.856	
	EA4	0.784		0.786	
	EA5	0.870		0.869	
	EA6	0.822		0.820	
	EA7	0.871		0.870	
Job satisfaction (JSAT)	JSAT1	0.820	0.598	0.821	0.741
	JSAT2	0.898		0.898	
	JSAT3	0.835		0.835	
	JSAT4	0.888		0.888	
	JSAT5	−0.151		Removed	
Job security (JSEC)	JSEC1	0.766	0.539	0.778	0.611
	JSEC2	0.846		0.842	
	JSEC3	0.473		Removed	
	JSEC4	0.752		0.761	
	JSEC5	0.732		0.731	
	JSEC6	0.776		0.793	
Life satisfaction (LS)	LS1	0.831	0.692	0.831	0.692
	LS2	0.859		0.859	
	LS3	0.845		0.845	
	LS4	0.879		0.880	
	LS5	0.739		0.738	
Optimism (OPT)	OPT1	0.795	0.553	0.803	0.648
	OPT2	0.840		0.849	
	OPT3	0.854		0.859	
	OPT4	0.444		Removed	
	OPT5	0.707		0.699	
Spiritual transcendence (ST)	SPT1	0.727	0.473	0.730	0.524
	SPT2	0.780		0.787	
	SPT3	0.728		0.732	
	SPT4	0.686		0.677	
	SPT5	0.751		0.755	
	SPT6	0.733		0.743	
	SPT7	0.736		0.742	
	SPT8	0.604		0.610	
	SPT9	0.336		Removed	

**Table 2:** Validity test result for Türkiye dataset (*N* = 456).

Variables	Indicators	Before indicators deletion		After indicators deletion	
		Outer loading (≥ 0.50 valid)	AVE (≥ 0.50 valid)	Outer loading (≥ 0.50 valid)	AVE (≥ 0.50 valid)
Earthquake anxiety (EA)	EA1	0.712	0.591	0.719	0.594
	EA2	0.730		0.737	
	EA3	0.813		0.813	
	EA4	0.692		0.698	
	EA5	0.717		0.727	
	EA6	0.848		0.841	
	EA7	0.851		0.845	
Job satisfaction (JSAT)	JSAT1	0.910	0.621	0.933	0.844
	JSAT2	0.937		0.954	
	JSAT3	−0.310		Removed	
	JSAT4	0.863		0.867	
	JSAT5	−0.747		Removed	
Job security (JSEC)	JSEC1	0.774	0.661	0.771	0.661
	JSEC2	0.835		0.835	
	JSEC3	0.802		0.803	
	JSEC4	0.828		0.829	
	JSEC5	0.774		0.775	
	JSEC6	0.861		0.862	
Life satisfaction (LS)	LS1	0.817	0.652	0.818	0.652
	LS2	0.831		0.830	
	LS3	0.857		0.856	
	LS4	0.810		0.812	
	LS5	0.714		0.713	
Optimism (OPT)	OPT1	0.671	0.474	0.706	0.578
	OPT2	0.788		0.790	
	OPT3	0.857		0.859	
	OPT4	−0.367		Removed	
	OPT5	0.654		0.672	
Spiritual transcendence (ST)	SPT1	0.835	0.442	0.836	0.562
	SPT2	0.877		0.878	
	SPT3	0.888		0.889	
	SPT4	−0.070		Removed	
	SPT5	0.676		0.676	
	SPT6	0.792		0.793	
	SPT7	0.562		0.558	
	SPT8	0.524		0.523	
	SPT9	−0.206		Removed	

The measurement of discriminant validity in current study was conducted through two stages of analysis. The first stage involved assessing the square root of AVE using the Fornell-Larcker Criterion, and these results were then confirmed through the *Heterotrait-Monotrait (HTMT) ratio* Values. The *Fornell-Larcker Criterion* test results on the Bali dataset (Table 3) and the Türkiye dataset (Table 4) affirm that the square root of *AVE* for each construct exceeds the square root values of *AVE* in other constructs. Subsequently, the *HTMT* test result on the Bali dataset (Table 5) and the Türkiye dataset (Table 6) confirm that each construct obtained an *HTMT* value of less than 0.90. These findings underline that no strong correlations were found between research constructs (Fornell and Larcker, 1981; Henseler et al., 2015; Henseler, 2017).

**Table 3:** Fornell-Larcker Criterion test results for Bali dataset (*N* = 457).

Variables	Earthquake anxiety	Job satisfaction	Job security	Life satisfaction	Optimism	Spiritual transcendence
Earthquake anxiety	0.830					
Job satisfaction	0.358	0.861				
Job security	0.289	0.615	0.782			
Life satisfaction	0.358	0.583	0.532	0.832		
Optimism	0.270	0.548	0.601	0.539	0.805	
Spiritual transcendence	0.364	0.573	0.491	0.452	0.520	0.724

**Table 4:** Fornell-Larcker Criterion test results for Türkiye dataset (*N* = 456).

Variables	Earthquake anxiety	Job satisfaction	Job security	Life satisfaction	Optimism	Spiritual transcendence
Earthquake anxiety	0.771					
Job satisfaction	−0.041	0.919				
Job security	−0.098	0.501	0.813			
Life satisfaction	−0.139	0.564	0.518	0.807		
Optimism	−0.112	0.543	0.569	0.613	0.760	
Spiritual transcendence	−0.039	0.409	0.283	0.470	0.550	0.749

**Table 5:** HTMT test results for Bali dataset (*N* = 457).

Variables	Earthquake anxiety	Job satisfaction	Job security	Life satisfaction	Optimism
Earthquake anxiety					
Job satisfaction	0.394				
Job security	0.327	0.713			
Life satisfaction	0.396	0.655	0.618		
Optimism	0.307	0.639	0.717	0.625	
Spiritual transcendence	0.404	0.651	0.567	0.511	0.613

**Table 6:** HTMT test results for Türkiye dataset (*N* = 456).

Variables	Earthquake anxiety	Job satisfaction	Job security	Life satisfaction	Optimism
Earthquake anxiety					
Job satisfaction	0.069				
Job security	0.090	0.551			
Life satisfaction	0.143	0.631	0.579		
Optimism	0.172	0.646	0.673	0.746	
Spiritual transcendence	0.091	0.453	0.318	0.528	0.692

### 4.2 Reliability assessment

Tables 7, 8 provide the outcomes of the internal consistency assessment utilizing two parameters: *Cronbach’s Alpha* and *Composite Reliability*. The results confirm the reliability of the data utilized in this study, as all parameters exceeded the threshold of 0.70. In the Bali dataset (Table 5), *Cronbach’s Alpha* values for all variables ranged from 0.817 to 0.924, while in the Türkiye dataset, (Table 4) the reliability scores ranged from 0.755 to 0.907. Additionally, based on the *composite reliability* parameters, scores ranging from 0.880 to 0.939 were obtained in the Bali dataset and between 0.844 to 0.942 in the Türkiye dataset.

**Table 7:** Reliability test results for Bali dataset (*N* = 457).

Variables	Cronbach’s Alpha	Composite reliability
	(≥ 0.70 reliable)	
Earthquake anxiety	0.924	0.939
Job satisfaction	0.883	0.920
Job security	0.841	0.887
Life satisfaction	0.888	0.918
Optimism	0.817	0.880
Spiritual transcendence	0.869	0.898

**Table 8:** Reliability test results for Türkiye dataset (*N* = 456).

Variables	Cronbach’s Alpha	Composite reliability
	(≥ 0.70 Reliable)	
Earthquake anxiety	0.898	0.911
Job satisfaction	0.907	0.942
Job security	0.897	0.921
Life satisfaction	0.866	0.903
Optimism	0.755	0.844
Spiritual transcendence	0.864	0.896

### 4.3 R-square test

Tables 9, 10 illustrate the outcomes of the *R^2^ test*. In the Bali dataset, the highest *R^2^* value was achieved for the job satisfaction construct, scoring 0.427. This indicates that 42.7% of the variance in job satisfaction can be explained by the spiritual transcendence and earthquake anxiety constructs. Subsequently, the job security construct attained an *R^2^* value of 0.406, suggesting that 40.6% of the variance in job security can be predicted by the earthquake anxiety and spiritual transcendence constructs. In the Türkiye dataset, the highest *R^2^* value was observed for the life satisfaction construct, reaching 0.411. This implies that 41.1% of the variance in life satisfaction can be accounted for by the spiritual transcendence and earthquake anxiety constructs. Additionally, the job security construct obtained an *R^2^* score of 0.322, indicating that 32.2% of the variance in job security can be predicted by the spiritual transcendence and earthquake anxiety constructs.

**Table 9:** *R^2^* test result for Bali dataset (*N* = 457).

Variables	R Square	R Square adjusted
Earthquake anxiety	0.132	0.130
Job satisfaction	0.432	0.427
Job security	0.411	0.406
Life satisfaction	0.376	0.371

**Table 10:** *R^2^* test result for Türkiye dataset (*N* = 456).

Variables	R Square	R Square adjusted
Earthquake anxiety	0.002	−0.001
Job satisfaction	0.322	0.316
Job security	0.328	0.322
Life satisfaction	0.416	0.411

### 4.4 Hypotheses testing

Tables 11–13 summarize the outcomes of direct, mediation, and moderation hypothesis testing. In the Bali dataset, seven hypotheses were accepted, while three were rejected. Conversely, in the Türkiye dataset, four hypotheses were accepted, and six were rejected. Firstly, the relationship between spiritual transcendence and earthquake anxiety was found to be positive and significant in the Bali dataset, with a coefficient value of 0.364, a *t*-statistic of 8.336 (> 1.65), and a *p*-value of 0.000 (< 0.05). However, this relationship was rejected in the Türkiye dataset, with a path coefficient of −0.039, a *t*-statistic of 0.417 (< 1.65), and a *p*-value of 0.339 (> 0.05). Secondly, the relationship between spiritual transcendence and job security was positive and significant in the Bali dataset, with a path coefficient of 0.225, a *t*-statistic of 4.328 (> 1.65), and a *p*-value of 0.000 (< 0.05).

**Table 11:** Hypotheses test result for Bali dataset (*N* = 457).

Hypotheses path		Original sample	*T* Statistics (≥ 1.65)	*p* values (≤ 0.05)
H1	Spiritual Transcendence -> Earthquake Anxiety	0.364	8.336	0.000
H2	Spiritual Transcendence -> Job Security	0.225	4.328	0.000
H3	Spiritual Transcendence -> Earthquake Anxiety -> Job Security	0.028	1.586	0.057
H4	Spiritual Transcendence -> Life Satisfaction	0.203	3.932	0.000
H5	Spiritual Transcendence -> Earthquake Anxiety -> Life Satisfaction	0.056	2.741	0.003
H6	Spiritual Transcendence -> Job Satisfaction	0.350	7.135	0.000
H7	Spiritual Transcendence -> Earthquake Anxiety -> Job Satisfaction	0.052	3.079	0.001
H8	Earthquake Anxiety*Optimism -> Job Security	0.028	0.757	0.225
H9	Earthquake Anxiety*Optimism -> Life Satisfaction	0.113	3.087	0.001
H10	Earthquake Anxiety*Optimism -> Job Satisfaction	−0.004	0.107	0.457

**Table 12:** Hypotheses test result for Türkiye dataset (*N* = 456).

Hypotheses path		Original sample	*T* statistics (≥ 1.65)	*p* values (≤ 0.05)
H1	Spiritual Transcendence -> Earthquake Anxiety	−0.039	0.417	0.339
H2	Spiritual Transcendence -> Job Security	−0.045	0.831	0.203
H3	Spiritual Transcendence -> Earthquake Anxiety -> Job Security	0.001	0.261	0.397
H4	Spiritual Transcendence -> Life Satisfaction	0.185	3.768	0.000
H5	Spiritual Transcendence -> Earthquake Anxiety -> Life Satisfaction	0.003	0.402	0.344
H6	Spiritual Transcendence -> Job Satisfaction	0.150	2.569	0.005
H7	Spiritual Transcendence -> Earthquake Anxiety -> Job Satisfaction	−0.001	0.107	0.457
H8	Earthquake Anxiety*Optimism -> Job Security	−0.034	0.893	0.186
H9	Earthquake Anxiety*Optimism -> Life Satisfaction	−0.088	2.343	0.010
H10	Earthquake Anxiety*Optimism -> Job Satisfaction	−0.092	2.188	0.015

**Table 13:** Hypotheses test result summary.

Hypotheses path		Bali dataset (*N* = 456)	Türkiye dataset (*N* = 457)
H1	Spiritual Transcendence -> Earthquake Anxiety	Supported	Not Supported
H2	Spiritual Transcendence -> Job Security	Supported	Not Supported
H3	Spiritual Transcendence -> Earthquake Anxiety -> Job Security	Not Supported	Not Supported
H4	Spiritual Transcendence -> Life Satisfaction	Supported	Supported
H5	Spiritual Transcendence -> Earthquake Anxiety -> Life Satisfaction	Supported	Not Supported
H6	Spiritual Transcendence -> Job Satisfaction	Supported	Supported
H7	Spiritual Transcendence -> Earthquake Anxiety -> Job Satisfaction	Supported	Not Supported
H8	Earthquake Anxiety*Optimism -> Job Security	Not Supported	Not Supported
H9	Earthquake Anxiety*Optimism -> Life Satisfaction	Supported	Supported
H10	Earthquake Anxiety*Optimism -> Job Satisfaction	Not Supported	Supported

Conversely, in the Türkiye dataset, this relationship was negative and not significant, with a coefficient value of −0.045, a *t*-statistic of 0.831 (< 1.65), and a *p*-value of 0.203 (> 0.05). Next hypothesis, the role of earthquake anxiety in mediating the relationship between spiritual transcendence and job security was found to be insignificant in both Bali and Türkiye datasets, as the *t*-statistic values of 1.586 (< 1.65) and 0.261 (< 1.65) with *p*-values of 0.057 (> 0.05) and 0.397 (> 0.05). Fourth, a positive and significant relationship between spiritual transcendence and life satisfaction was found in both datasets. In the Bali dataset, the path coefficient value was 0.203, compared to 0.185 in the Türkiye dataset. Additionally, both datasets obtained *t*-statistic values of 3.932 (> 1.65) and 3.768 (> 1.65) and both *p*-values of 0.000 (< 0.05).

The results of the fifth hypothesis test indicate that earthquake anxiety serves as a mediating variable in the relationship between spiritual transcendence and life satisfaction in the Bali dataset. This is supported by a *t*-statistic value of 2.741 (> 1.65) with a *p*-value of 0.003 (< 0.05). However, in the Türkiye dataset, earthquake anxiety fails as a mediating variable, as evidenced by a *t*-statistic value of 0.402 (< 1.65) with a *p*-value of 0.344 (> 0.05). For the sixth hypothesis, a positive and significant relationship between spiritual transcendence and job satisfaction was observed in both datasets.

In the Bali dataset, a higher path coefficient value of 0.350 was obtained compared to the Türkiye dataset, which had a coefficient value of 0.150. Furthermore, the *t*-statistic values were above 1.65, and the *p*-values were below 0.05 in both datasets. Hypothesis seven confirms that earthquake anxiety does not mediate the relationship between spiritual transcendence and job satisfaction in the Türkiye dataset. This is evident from the *t*-statistic value obtained at 0.107 (< 1.65) with a *p*-value of 0.457 (> 0.05). Conversely, in the Bali dataset, earthquake anxiety successfully acts as a mediating variable, as indicated by the *t*-statistic value of 3.079 (> 1.65) and *p*-values of 0.001 (< 0.05).

The eighth to tenth hypotheses represent the results of the moderation test of the optimism construct, with only the ninth hypothesis being accepted in both research datasets. These findings suggest that optimism moderates the relationship between earthquake anxiety and life satisfaction, supported by *t*-statistic values exceeding 1.65 and *p*-values below 0.05. In the Bali dataset, optimism strengthens this relationship with a positive path coefficient of 0.113. Conversely, in the Türkiye dataset, optimism weakens the relationship with a negative path coefficient of 0.088.

However, the eighth hypothesis was rejected in both datasets, indicating that optimism does not moderate the relationship between earthquake anxiety and job security. This is supported by *t*-statistics below 1.65 and *p*-values exceeding 0.05. Finally, the tenth hypothesis reveals that optimism weakens the relationship between earthquake anxiety and job satisfaction in the Türkiye dataset, with a path coefficient of −0.092, a *t*-statistic of 2.188 (> 1.65), and a *p*-value of 0.015 (< 0.05). In contrast, in the Bali dataset, optimism does not significantly moderate this relationship, as indicated by a *t*-statistic of 0.107 (< 1.65) and a *p*-value of 0.457 (> 0.05).

## 5 Discussion

Spirituality, which has its origins in people’s early development, may be a potent source of both rejection and denigration as well as hope and affirmation from the perspective of life span development (Wolfe et al., 2003; Millstein et al., 2019). Previous studies revealed the importance of spirituality in combatting with the stress caused by disasters (Van Tongeren et al., 2020; Zhang et al., 2022). With the assumption that both Balinese and Turkish cultures are highly spiritual, we wanted to investigate the potential negative impact of spiritual transcendence on earthquake anxiety. In general, higher levels of spirituality have a soothing effect on death anxiety levels of individuals (Pandya and Kathuria, 2021). However, the results did not align with our expectations.

Taking earthquake anxiety, as a form of death anxiety, the relationship between spiritual transcendence and earthquake anxiety was positive in the Balinese case and negative but weak in the Turkish case. The Balinese case make us think that sensibility, fear of death and emotional sensitiveness coming from Balinese religion (Jayanti et al., 2024) may be creating over sensitiveness about issues related to death and detrimental events and disasters evoking earthquake anxiety. On the one hand, according to the results of the study, we noticed that participants in Turkish sample are not as spiritual as we have expected so the weak effect of spiritual transcendence on earthquake anxiety is understandable. The extant literature supports this by showing the weak effect of spirituality on anxiety in Turkish case (Çalışkan and Gürses, 2024) and supports the positive effect of spiritual transcendence in Balinese context (Suardana et al., 2023).

According to existing literature, when people experience spiritual transcendence, they become more positive about life (Elkady, 2019), which helps them feel more secure in their jobs. The positive relationship between spiritual transcendence and job security was confirmed in the Balinese case, but not in the Turkish case. Actually, the findings indicate that Turkish participants’ spiritual transcendence has a lesser impact on their daily lives than the Balinese sample. We consider this as a hint that that spirituality is not a major life issue that affects people’s daily lives. Moreover, hypothesis related to the mediator effect of earthquake anxiety and job security is rejected in both samples revealing no significant effect of earthquake anxiety as a mediator between spiritual transcendence and job security relationship. This may be because of the fact that job security is effected by many other factors like macroeconomic shocks (Johnston et al., 2020), political turmoil (El Khoury-Malhame et al., 2023) and pandemics (Baykal et al., 2023), hence the effects combined effect of spiritual transcendence and earthquake anxiety may be ignorable. Especially for Turkish case, economic problems experienced in recent years and political turmoil in middle east may be more effective than other factors in creating the sense of job security or insecurity. (Üngüren et al., 2024; Yıldırımhan et al., 2024).

On the one hand, although there are significant proofs for the positive effect of spiritual transcendence on life satisfaction (Maier and Surzykiewicz, 2020; Yaden et al., 2022). Dorais and Gutierrez (2021) empirically revealed the positive impact of spiritual transcendence on resilience levels of individuals which boosts life satisfaction. In our study this is validated only for Balinese sample but not for Turkish sample. This can be because the fact that in the Turkish spirituality in not experienced very deeply in daily lives (Yapıcı, 2020) of many individuals as in the case in Balinese example. Furthermore, a positive and statistically significant relationship between spiritual transcendence and job satisfaction has been observed in both samples, confirming the findings of studies related to the positive relationship between individual level spirituality and job satisfaction in the extant literature (Rashidin et al., 2020; Asutay et al., 2022), however this study is novel in the point that it tested spiritual transcendence rather than individual spirituality. It is interesting that, in our comparative study, earthquake anxiety has ended up being a mediator between spiritual transcendence and job satisfaction in the Balinese sample but not in the Turkish sample, which may be a hint that the earthquake anxiety affects Balinese people’s peace of mind at work more strongly in the Balinese sample compared to the Turkish sample, which is why earthquake anxiety shadows the effect of spiritual transcendence on job satisfaction is more significant in Balinese case.

Moreover, when the moderator effect of optimism is tested, it is discovered that it acts as a moderator in both samples in the relationship between earthquake anxiety and life satisfaction, supporting the importance of optimism in shaping our perspectives in anxiety-provoking events (Rajandram et al., 2011; Fasano et al., 2020). However, in the Balinese sample, optimism strengthens the relationship with a weak positive path, whereas in the Turkish sample, optimism weakens the relationship with a relatively strong negative path. Remembering the empirical proofs that Turkish people are inclined to be rather pessimistic (Yetim et al., 2024), this results are understandable an opitimistic approach can create a greater added value in a pessimistic culture compared to the situation in a peaceful and more positive culture like Bali (Ardhana, 2020).

Based on the findings above, we may conclude that in the Balinese sample, both earthquake anxiety and the effect of spiritual transcendence on people’s satisfaction levels and perceptions of job security are stronger than that are in the Turkish group. As a result, we can conclude that Turkish people experience earthquake anxiety differently than Balinese people. Moreover, spirituality has a more significant impact on the Balinese sample than the Turkish sample. This could be due to the fact that religion continues to have a significant impact on modern Bali culture. However, the modernization period is experienced in a more modern way in Turkish, making the Turkish community more secular, resulting in a significant lack of spiritual knowledge (Asanatuci, 2023).

## 6 Conclusion

In our hypothesis, we had assumed a negative relationship between spiritual transcendence and earthquake anxiety. However, our findings revealed that this relationship is positive in the Balinese case is non-existent in the Turkish sample. Similarly, whereas there is a positive relationship between spiritual transcendence and job security in Balinese case, this positive relationship does not exist in Turkish case. Moreover, against our assumptions, we could not find the traces of mediator effect of earthquake anxiety in the relationship between spiritual transcendence and job satisfaction in both contexts. On the one hand, regarding the overall satisfaction in life we investigated the relationship between spiritual transcendence and life satisfaction and we ended up with positive relationships between these two factors in Balinese samples but not in Turkish sample. However, a positive and significant relationship between spiritual transcendence and job satisfaction was observed in both datasets. Similarly, earthquake anxiety acts as a mediator in the relationship between spiritual transcendence and job satisfaction in Balinese sample but not in Turkish sample. Furthermore, optimism moderates the relationship between earthquake anxiety and life satisfaction in both samples. Whereas only in Turkish sample, optimism moderated the relationship between earthquake anxiety and job satisfaction.

This study is novel in comparing two significantly different but spiritually sensitive cultures, one of which is dominantly adopts a monotheist religion and the other a polytheist religion. But to increase the representativeness of the study different countries from different monotheist religions and polytheist religions can be added. Moreover it increase the representatives of the study if the profile of participants are limited more specifically.

## 7 Managerial implications and further studies

While designing this study, the sample was drawn from people living in Bali and earthquake-prone regions in Türkiye. To make the research results more reliable, the model can be repeated in future studies on earthquake victims who have recently experienced the earthquake, as well as tested in other countries. Our research has shown that the effect of spiritual transcendence and earthquake anxiety on life and job satisfaction in general is much more evident in Bali than in Türkiye, which has a very different religious practice in cultural terms, and provides an important opportunity to understand the differences in the reflections of spiritual perspectives in two different societies.

## Data availability statement

The original contributions presented in the study are included in the article/supplementary material, further inquiries can be directed to the corresponding author.

## Ethics statement

Ethical review and approval was not required for the study on human participants in accordance with the local legislation and institutional requirements. Written informed consent from the patients/participants or patients/participants’ legal guardian/next of kin was not required to participate in this study in accordance with the national legislation and the institutional requirements.

## Author contributions

NS: Writing – review & editing, Writing – original draft, Visualization, Validation, Supervision, Software, Resources, Project administration, Methodology, Investigation, Funding acquisition, Formal analysis, Data curation, Conceptualization. EB: Writing – review & editing, Writing – original draft, Visualization, Validation, Supervision, Software, Resources, Project administration, Methodology, Investigation, Funding acquisition, Formal analysis, Data curation, Conceptualization. IB: Writing – review & editing, Writing – original draft, Visualization, Validation, Supervision, Software, Resources, Project administration, Methodology, Investigation, Funding acquisition, Formal analysis, Data curation, Conceptualization. CM: Writing – review & editing, Writing – original draft, Visualization, Validation, Supervision, Software, Resources, Project administration, Methodology, Investigation, Funding acquisition, Formal analysis, Data curation, Conceptualization. HY: Writing – review & editing, Writing – original draft, Visualization, Validation, Supervision, Software, Resources, Project administration, Methodology, Investigation, Funding acquisition, Formal analysis, Data curation, Conceptualization.
